# Interplay of intrinsic and network heterogeneity in strongly recurrent spiking networks

**DOI:** 10.1186/1471-2202-16-S1-P150

**Published:** 2015-12-18

**Authors:** Cheng Ly

**Affiliations:** 1 Department of Statistical Sciences and Operations Research, Virginia Commonwealth University, Richmond, VA 23284, USA

## 

Heterogeneity has recently gained a lot of attention and it is becoming more apparent that it is a crucial feature in neural processing [1-5]. Despite its importance, this realistic physiological feature has traditionally been neglected in theoretical studies of cortical neural networks. A common reason is that mean-field descriptions of noisy cortical networks are high dimensional and generally intractable. Although heterogeneous spiking neural networks have recently been studied theoretically [5-8], there is still a lot unknown. In particular, combining network heterogeneity [9] and intrinsic heterogeneity [10] have yet to be considered simultaneously despite the fact that both are known to exist and likely have significant roles in neural network dynamics.

To this end, we study a recurrently coupled spiking network of leaky integrate-and-fire (LIF) neurons consisting of excitatory and inhibitory neurons. The intrinsic heterogeneity is modeled by varying the voltage threshold for spiking [5], and the network heterogeneity is modeled by different conductance strengths (partially motivated by recent results [11], both excitatory and inhibitory conductances are scaled so each neuron has a different level of balanced input). Unsurprisingly, we find that when either intrinsic or network heterogeneity is increased, the response heterogeneity also increases (i.e., the range of the average firing rate of the excitatory neurons also increases). However, for a fixed level of both forms of heterogeneity, the network robustly exhibits a wide range of response heterogeneity that strongly depends on the relationship between intrinsic and network heterogeneity. This coupled network is difficult to analyze because it is stochastic, heterogeneous, and high dimensional with alpha function synapses and colored external noisy input. With combination of Monte Carlo simulations and augmented mean-field theory based partially on methods in [12-15], we provide analytic explanations to account for the observed phenomena. Our work gives insight for how these two forms of heterogeneity interact in a generic recurrent spiking network that may be applicable to many areas of the cortex.
